# Autopsy of COVID-19 patients in China

**DOI:** 10.1093/nsr/nwaa123

**Published:** 2020-06-06

**Authors:** Xiu-Wu Bian, The COVID-19 Pathology Team, Xiao-Hong Yao, Yi-Fang Ping, Shicang Yu, Yu Shi, Tao Luo, Zhi-Cheng He, Rui Tang, Cong Chen, Wen-Juan Fu, Hongyan Zhang, Hua-Rong Zhang, Dong-Fang Xiang, Qing-Rui Li, Xuequan Huang, Tingyuan Li, Pengnan Zhao, Chaofu Wang, Xiaochun Fei, Jun Cai, Lei Zhao, Heng Zhang, Zhenghua Liu, Liang Liu, Guoping Wang, Xiu Nie, Yiwu Zhou, Liang Ren, Qian Liu, Yi Wang, Qilin Ao, Xi Wang, Yaqi Duan, Jiansha Li, Jin Xiong, Sanpeng Xu, Jie Zhang, Sizhe Huang, Ming Yang, Bo Huang, Xiang Li, Lixu Peng, Pan Xi, Xiong Hua, Hua Su, Sihua Wangcheng, Cheng Yu, Haibo Wu, Heng Li, Yong Ren, Xinwei Chen, Liwei Liang, Zongxing Zhang, Rong Chen, Fei Deng, Guoqiang Qu, Rongshuai Wang, Yunyun Wang, Xiaowei Zhou, Fusheng Wang, Jingmin Zhao

**Affiliations:** The initiating member of the COVID-19 Pathology Team, Institute of Pathology & Southwest Cancer Center, Southwest Hospital, Third Military Medical University (Army Medical University), China; Southwest Hospital, Third Military Medical University, China; Southwest Hospital, Third Military Medical University, China; Southwest Hospital, Third Military Medical University, China; Southwest Hospital, Third Military Medical University, China; Southwest Hospital, Third Military Medical University, China; Southwest Hospital, Third Military Medical University, China; Southwest Hospital, Third Military Medical University, China; Southwest Hospital, Third Military Medical University, China; Southwest Hospital, Third Military Medical University, China; Southwest Hospital, Third Military Medical University, China; Southwest Hospital, Third Military Medical University, China; Southwest Hospital, Third Military Medical University, China; Southwest Hospital, Third Military Medical University, China; Southwest Hospital, Third Military Medical University, China; Southwest Hospital, Third Military Medical University, China; Southwest Hospital, Third Military Medical University, China; Shanghai Jiao Tong University, China; Shanghai Jiao Tong University, China; Shanghai Jiao Tong University, China; Shanghai Jiao Tong University, China; Shanghai Jiao Tong University, China; Shanghai Jiao Tong University, China; Huazhong University of Science & Technology, China; Huazhong University of Science & Technology, China; Huazhong University of Science & Technology, China; Huazhong University of Science & Technology, China; Huazhong University of Science & Technology, China; Huazhong University of Science & Technology, China; Huazhong University of Science & Technology, China; Huazhong University of Science & Technology, China; Huazhong University of Science & Technology, China; Huazhong University of Science & Technology, China; Huazhong University of Science & Technology, China; Huazhong University of Science & Technology, China; Huazhong University of Science & Technology, China; Huazhong University of Science & Technology, China; Huazhong University of Science & Technology, China; Huazhong University of Science & Technology, China; Huazhong University of Science & Technology, China; Huazhong University of Science & Technology, China; Huazhong University of Science & Technology, China; Huazhong University of Science & Technology, China; Huazhong University of Science & Technology, China; Huazhong University of Science & Technology, China; Huazhong University of Science & Technology, China; Huazhong University of Science & Technology, China; University of Science and Technology of China, China; University of Science and Technology of China, China; Central Theater General Hospital, China; Central Theater General Hospital, China; Central Theater General Hospital, China; Academy of Military Sciences, China; Wuhan Jinyintan Hospital, China; Wuhan Institute of Virology, Chinese Academy of Sciences, China; Hubei Chongxin Forensic Center, China; Hubei Chongxin Forensic Center, China; Hubei Chongxin Forensic Center, China; Hubei Chongxin Forensic Center, China; The Fifth Medical Center, General Hospital of PLA, China; The Fifth Medical Center, General Hospital of PLA, China

Autopsy is of paramount significance for comprehensively understanding the pathological features of COVID-19, clarifying viral invading routes and distribution in the body, to benefit prevention and treatment. Under the forceful leadership of the Chinese government, the battle against the COVID-19 pandemic has achieved a decisive victory at the present time. In response to the pandemic, pathologists in China have actively participated in COVID-19-patient autopsy and have made important discoveries in both the etiology and pathology of COVID-19. These efforts have strongly supported accuracy in diagnosis and treatment [1–4], and thus have significantly contributed to victory over the COVID-19 pandemic.

## GENERAL PATHOLOGY OF COVID-19-PATIENT AUTOPSY

Autopsy is vital for the final diagnosis of COVID-19. Upon the outbreak of COVID-19, >60 pathologists and technicians assembled a COVID-19 Pathology Team in Wuhan and Chongqing, China. We took the initiative to perform patient autopsy in compliance with the relevant Chinese laws. Pathologists from designated special institutions including the Third Military Medical University (TMMU), Shanghai Jiao Tong University, Huazhong University of Science and Technology, University of Science and Technology of China (USTC) and several other medical institutions were designated as special groups and conducted systematic autopsy and percutaneous biopsy on patients who had succumbed to COVID-19.

As of 22 April 2020, the COVID-19 Pathology Team has performed systematic autopsies on 37 COVID-19 cases. The team of the TMMU (Army Medical University), together with colleagues from Shanghai Jiao Tong University Medical School and Ruijin Hospital, the First Affiliated Hospital of the USTC and the Central Theater General Hospital, completed 27 cases from Wuhan Jin-yintan Hospital, Wuhan Huoshenshan Hospital, Tongji Zhongfaxincheng Hospital and Taikang Tongji Hospital. The team of Huazhong University of Science and Technology Tongji Medical School completed 10 cases from Wuhan Jinyintan Hospital and Wuhan Central Hospital. In addition, percutaneous multiple-organ biopsy (‘minimally invasive autopsy’) was carried out in 54 cases. Among them, the team of Wuhan Union Hospital completed 30 cases from Wuhan Union Hospital West District and the team of TMMU completed 13 cases from Chongqing Three Gorges Central Hospital, Wuhan Tongji Hospital Zhongfaxincheng Hospital and Wuhan Huoshenshan Hospital. The team from Wuhan Tongji Hospital completed 9 cases from Wuhan Tongji Hospital Zhongfaxincheng Hospital and the team of the Fifth Clinical Medical Center of the PLA General Hospital completed two cases. Altogether, a total of 91 COVID-19 patients were pathologically inspected and diagnosed, which may represent the largest number of autopsy cases with the most comprehensive examination in the world thus far.

Furthermore, our systematic autopsy has established a basis for collaboration among a number of pathological institutions during the COVID-19 pandemic in China. During the process, anatomic pathologists and technicians also collaborated with engineers to establish the biosafety platforms for the autopsy of COVID-19 patients. Governmental laws and regulations played an important role in mobilizing a rapid response to this major public-health emergency.

## MAJOR FINDINGS FROM THE AUTOPSY

**Figure 1.**
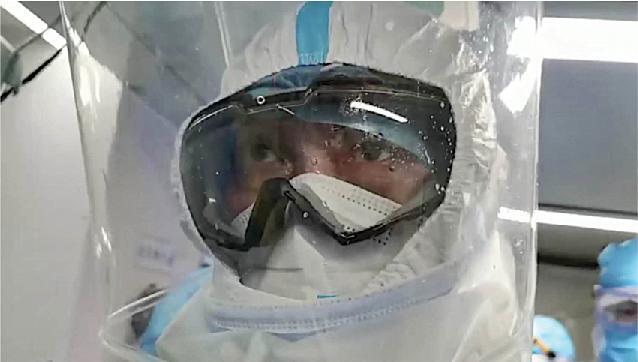
A Chinese pathologist performing a COVID-19 autopsy. Courtesy of Hongyan Zhang, Southwest Hospital, Chongqing, China.

**Figure 2.**
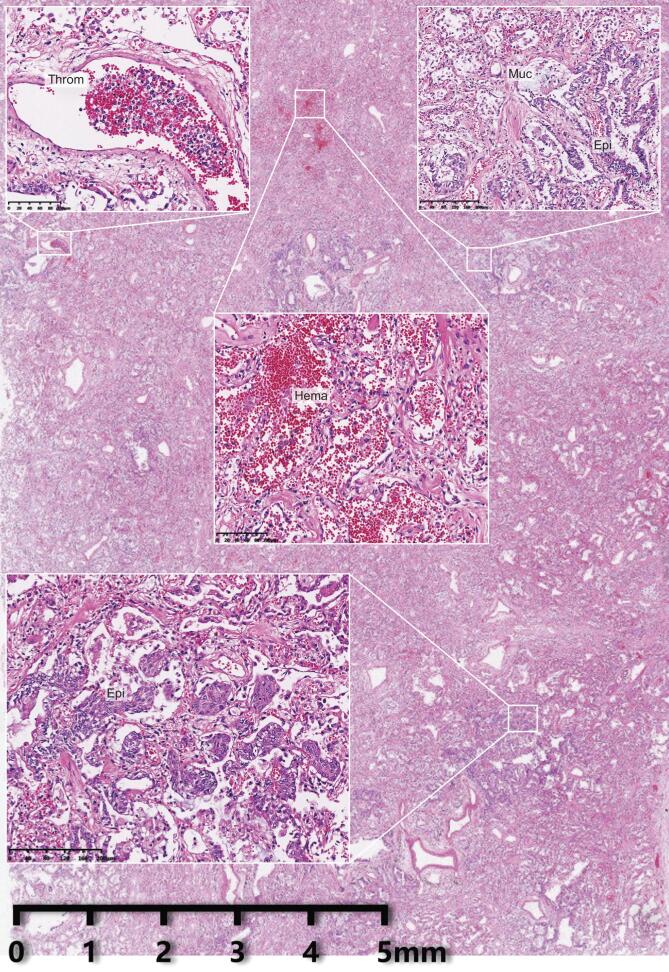
An overview of pulmonary histopathological changes in COVID-19. Exudate, epithelial (Epi) proliferation, thrombosis (Throm), mucus plugs (Muc) and hemorrhage (Hema) are seen in an affected pulmonary lobe.

The Pathology Team has performed intensive pathological diagnosis and research at organ, tissue, cell, ultrastructural and molecular levels (Fig. 1). We discovered that SARS-CoV-2 infection causes injuries in multiple organs and tissues with prominent and extensive pulmonary lesions. The pathological characteristics of pulmonary lesions caused by SARS-CoV-2 infection were similar to those from SARS infection but differences have been noticed. Intensive spatial and temporal heterogeneity of pulmonary lesions was observed in individual patients (Fig. 2). Pathological changes in the respiratory system were most significant. Trachea and bronchus manifested mucosa congestion, increased secretion and focal epithelial exfoliation. Changes in pulmonary parenchyma presented with differences in extent and distribution. Under light microscopy, the parenchymal areas contained diffuse alveolar damage and exudative inflammation. Serous and fibrin exudate filled alveolar spaces, and hyaline membrane formation could be seen. The infiltrating leukocytes in the alveoli were mainly monocytes and macrophages. Type II pneumocyte hyperplasia and focal pneumocyte exfoliation were clearly observed. Some areas of bronchial mucosa manifested epithelial exfoliation, mucin accumulation and mucin-plug formation. A few alveoli showed excessive inflation, septal rupture or cystic cavity formation. Focal pulmonary hemorrhage and consequential hemorrhagic infarction or necrosis were observed. Pulmonary vessels showed vasculitis, thrombosis (mixed thrombi and hyaline thrombi) and thromboembolism. Exudate organization (also termed pulmonary carnification) and pulmonary interstitial fibrosis were observed in cases with long disease duration (see pulmonary complications in Box 1). Under electron microscopy, coronavirus particles were observed in the cytoplasm of tracheal and bronchial mucosa epithelia and alveolar type II pneumocytes. Immunohistochemical staining demonstrated that some tracheal and bronchial mucosa epithelia and type II alveolar epithelial cells and the infiltrating macrophages, which express angiotensin converting enzyme 2 (ACE2), were positive for SARS-CoV-2 protein. The trachea and pulmonary tissues (except some lung lobes) were positive for SARS-CoV-2 nuclear acid. Functionally, alveolar damage, exudation, interstitial inflammation and extensive thrombosis constituted a cause for ventilatory disorder. Airway epithelial hyperplasia, exfoliation and mucus congestion increased ventilation obstruction, especially in the small airway. Altogether, these changes were considered as a pathological basis for lethal respiratory failure.

**Box 1.** Pulmonary complications found in systematic autopsy of COVID-19 patients.

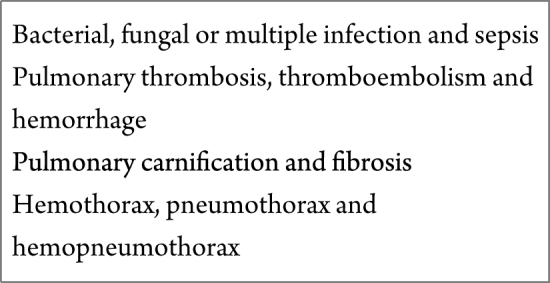

SARS-CoV-2 infection affected multiple organs to a different extent of acute injuries (see infected multiple organs in Table 1). We noticed apparent lesions in lymphatic hematopoietic organs. Lymphocytes, especially CD4^+^ and CD8^+^ T-cells, were significantly reduced in the spleen and lymph nodes. Lymphocyte degeneration, necrosis and macrophage proliferation were observed. Hemorrhage and anemic infarction were often found in the spleens. Bone marrow in most cases contained reduced erythroid cells, myeloid cells and megakaryocytes, whereas a few cases also showed apparent hyperplasia. Myocardia displayed cell degeneration, scattered necrosis, interstitial edema and mild infiltration of monocytes, lymphocytes and/or neutrophils. Multiple post-mortem regions showed tunica intima inflammation, thrombosis, anemic infarct (in organs such as the spleen, kidneys and heart) and hemorrhagic infarction (in organs such as the lungs and liver). The liver showed hepatocyte degeneration, spotty necrosis and piecemeal, bridging or massive necroses with neutrophil infiltration. Liver sinusoid congestion and infiltration of lymphocytes and monocytes in the portal areas were observed. The majority of the gallbladders were highly filled and mucosa epithelia were exfoliated. The kidneys presented with hyperemia, segmental hyperplasia or necrosis in glomeruli and protein exudate in glomerular-capsule chambers. Proximal tubules manifested epithelial degeneration, focal necrosis and exfoliation. Hyaline cast was often detected in distal tubules. Renal interstitial tissues displayed congestion, mild inflammatory-cell infiltration and fibrous hyperplasia. Adrenal-gland cortex degeneration, focal hemorrhage and necrosis were noticed. Pancreatic islet cell degeneration and dissolving were occasionally detected. Esophagus, stomach and intestine mucosa epithelia manifested different extents of degeneration, necrosis and exfoliation. Testes showed various degrees of spermatogenic cell reduction and injury. Brain hyperemia and edema, partial neuron degeneration and ischemic changes were detected. Some cases showed neuronophagia, inflammatory-cell infiltration in perivascular regions and focal cerebral infarction. A few cases manifested brain herniation. Through qRT-PCR-based virus nuclear acid detection, electron microscopy and immunohistochemical staining, SARS-CoV-2 was detected in the hilar lymph nodes, spleen, heart, liver, gallbladder, kidneys, stomach, breast, testes, skin, and nasopharyngeal and oral mucosa (Table 1).

**Table 1.** The infectious multi-organs in systematic autopsy of COVID-19.

	Detection methods		
	PCR	IHC	TEM
Respiratory system			
Nasopharynges	+	+	+
Trachea	+	+	+
Lungs	+	+	+
Cardiovascular system			
Heart	+	+	+
Blood vessels	+	+	+
Immune system			
Spleen	+	+	+
Lymph nodes	+	+	ND
Digestive system			
Liver	+	+	ND
Esophagus	+	+	ND
Stomach	+	+	ND
Small intestine	+	+	+
Colon	+	+	ND
Gallbladder	+	+	ND
Pancreas	+	ND	ND
Salivary glands	+	+	ND
Urinary and genital systems			
Kidneys	+	+	+
Ureter	+	+	ND
Bladder	+	+	ND
Breasts	+	+	ND
Uterus	+	+	ND
Ovary	+	+	+
Testes	+	+	+
Endocrine system			
Thyroid glands	+	+	ND
Adrenal glands	+	+	ND
Nervous system			
Cerebrum	+/–	+/–	ND
Cerebellum	+/–	+/–	ND

PCR, polymerase chain reaction; IHC, immunohistochemistry; TEM, transmission electron microscopy; ND, no detection; +, positive result; +/–, positive result to be confirmed.

We also noticed that the cadavers, especially the elders, had primary health conditions such as (i) respiratory diseases: chronic bronchitis, bronchiectasis, pulmonary fibrosis, emphysema and pulmonary bullae and other conditions; (ii) cardiovascular diseases: atherosclerosis in large and medium arteries, aneurysm rupture, cardiac hypertrophy, aged myocardial infarction, arteriolosclerosis and other conditions; (iii) digestive diseases: viral hepatitis and liver cirrhosis, chronic gastritis, calculous cholecystitis and other conditions; (iv) urinary diseases: chronic glomerulonephritis, renal cysts, chronic cystitis, benign hyperplasia of the prostate and other conditions; (v) hematologic abnormalities: bone-marrow steatosis and fibrosis and other conditions; (vi) brain lesions: aged cerebral infarction, lipofuscin accumulation and amyloid deposition. These existing conditions combined with the acute damage caused by SARS-CoV-2 constituted the pathological basis for multiple organ dysfunction syndrome, with more serious consequences from lesions in the lungs, heart, kidneys and liver. The major cause of death shown by the autopsy cases with COVID-19 appeared to be multiple organ dysfunction syndrome, especially acute respiratory distress syndrome. Some autopsy cases died from complications including severe pulmonary infections (bacteria, fungi or multiple infection), pulmonary embolism or hemorrhagic shock.

## CLINICAL APPLICATIONS OF COVID-19 AUTOPSY FINDINGS

Autopsy findings from COVID-19 patients have been applied to the clinic for improved diagnosis and treatment of the disease. Pathologists in the autopsy team and clinical experts discussed the pathological results and reached consensus on the pathological changes in COVID-19. The COVID-19 Diagnosis and Treatment Program (Trial Seventh Version) was issued, which fills the vacancy in the pathology for the diagnosis and treatment specifications of COVID-19. The program embodies important enhancements and adjustments in the treatment of COVID-19, such as promoting the establishment of a multi-organ support model, further strengthening the refinement of respiratory-function management, and emphasizing the assessment and protection of immune function.

The post-mortem discloses visible damage in the lungs and immune organs of COVID-19 patients. Since some immune organs are SARS-CoV-2-positive with spleen atrophy, the immune organs may be another target organ by virus infection in addition to the lungs, which emphasizes that clinical diagnosis and treatment should consider damage to the immune system. We found that most of the deceased COVID-19 cases had mucous plugs in the alveoli and deep small airways, which might severely affect ventilation. The mechanisms of the infection course and prevention measures are the subject of further investigation. In COVID-19 patients, pulmonary fibrosis occurred early and widely, suggesting the requirement for early measures on pulmonary fibrosis. In addition, the hyperplasia of pulmonary epithelial cells and the elevation of plasma carcinoembryonic antigen in some patients also require studies on the cause and follow-up. In some deceased cases, nasopharyngeal swab test showed negative viral nucleic acid, but positive in other organs [5], suggesting that the current standard swab test for SARS-CoV-2 as an indicator for clinical cure and hospital discharge may not be adequate. The detection of nuclear acids of SARS-CoV-2 in multiple organs of patients with severe conditions implies that the existence of SARS-CoV-2 and related organ damage might persist throughout the disease course even in patients with a longer term of disease. Preliminary findings of autopsy pathology revealed SARS-CoV-2 viral particles in multiple organs of several cases, suggesting a variety of viral-transmission routes such as the digestive tract, skin and various body fluids (saliva, urine, semen, sweat and milk, etc.). These issues merit further investigation.

## Notes

The other team members include Xiao-Hong Yao, Yi-Fang Ping, Shicang Yu, Yu Shi, Tao Luo, Zhi-Cheng He, Rui Tang, Cong Chen, Wen-Juan Fu, Hongyan Zhang, Hua-Rong Zhang, Dong-Fang Xiang, Qing-Rui Li, Xuequan Huang, Tingyuan Li, Pengnan Zhao (Southwest Hospital, Third Military Medical University, China)

Chaofu Wang, Xiaochun Fei, Jun Cai, Lei Zhao, Heng Zhang, Zhenghua Liu (Shanghai Jiao Tong University, China)

Liang Liu, Guoping Wang, Xiu Nie, Yiwu Zhou, Liang Ren, Qian Liu, Yi Wang, Qilin Ao, Xi Wang, Yaqi Duan, Jiansha Li, Jin Xiong, Sanpeng Xu, Jie Zhang, Sizhe Huang, Ming Yang, Bo Huang, Xiang Li, Lixu Peng, Pan Xi, Xiong Hua, Hua Su, Sihua Wangcheng, Cheng Yu, (Huazhong University of Science & Technology, China)

Haibo Wu, Heng Li (University of Science and Technology of China, China)

Yong Ren, Xinwei Chen, Liwei Liang (Central Theater General Hospital, China)

Zongxing Zhang (Academy of Military Sciences, China)

Rong Chen (Wuhan Jinyintan Hospital, China)

Fei Deng (Wuhan Institute of Virology, Chinese Academy of Sciences, China)

Guoqiang Qu, Rongshuai Wang, Yunyun Wang, Xiaowei Zhou (Hubei Chongxin Forensic Center, China)

Fusheng Wang, Jingmin Zhao (The Fifth Medical Center, General Hospital of PLA, China)

## References

[1] Zhu N, Zhang D, Wang W et al. N Engl J Med 2020; 382: 727–33. 31978945 10.1056/NEJMoa2001017 PMC7092803

[2] Wang D, Hu B, Hong C et al. JAMA 2020; 323: 1061–9. 32031570 10.1001/jama.2020.1585 PMC7042881

[3] Yao XH, Li TY, He ZC et al. Zhonghua Bing Li Xue Za Zhi 2020; 49: 411–7. 32172546 10.3760/cma.j.cn112151-20200312-00193

[4] Xu Z, Shi L, Wang Y et al. Lancet Respir Med 2020; 8: 420–2. 32085846 10.1016/S2213-2600(20)30076-X PMC7164771

[5] Yao XH, He ZC, Li TY et al. Cell Res 2020; 30: 541–3. 32346074 10.1038/s41422-020-0318-5 PMC7186763

